# Release of Cytokines in the Peritoneal Fluid of C57BL/6 Mice After *Bothrops jararaca* and *Bothrops atrox* Venom Injection

**DOI:** 10.3390/toxins17040164

**Published:** 2025-03-26

**Authors:** Adriana da Silva Fernandes Ribas, Kemily Stephanie de Godoi, Sávio Stefanini Sant’Anna, Marisa Maria Teixeira da Rocha, Wilmar Dias da Silva

**Affiliations:** 1 Immunochemistry Laboratory, Butantan Institute, São Paulo 05503-900, SP, Brazil; ribaslogia@gmail.com (A.d.S.F.R.); kemilysgodoi@gmail.com (K.S.d.G.); 2 Herpetology Laboratory, Butantan Institute, São Paulo 05503-900, SP, Brazil; savio.santanna@butantan.gov.br (S.S.S.); marisa.rocha@butantan.gov.br (M.M.T.d.R.)

**Keywords:** snake venom, inflammation, tropical disease, inflammatory response

## Abstract

The release of cytokines in the peritoneal fluid after stimulation with *Bothrops atrox* and *Bothrops jararaca* venoms is a crucial process in the inflammatory response triggered by these venoms. The toxins present in the venoms of snakes from the *Bothrops* genus induce a complex inflammatory response, which includes the production and release of pro-inflammatory cytokines such as TNF-α, IFN-γ, IL-6, IL-10, IL-1β, chemokines like GM-CSF, MCP-1, and the mast cell degranulation marker MCPT-1. These cytokines play a central role in amplifying inflammation, recruiting leukocytes, and increasing vascular permeability, resulting in edema, pain, and tissue damage at the inoculation site. Peritoneal fluid is commonly used in experimental studies to investigate local inflammatory responses, allowing for the evaluation of the dynamics of inflammatory molecule release. In this study, we used female C57BL/6 mice and observed that *Bothrops atrox* venom induced a significantly more intense inflammatory response compared to *Bothrops jararaca* venom. Specifically, *Bothrops atrox* venom led to a higher release of TNF-α and an increase in MCP-1 levels in peritoneal fluid when compared to *Bothrops jararaca* venom. These changes resulted in a more pronounced inflammatory condition, with increased leukocyte recruitment in the *Bothrops atrox* group. Understanding the cytokine profile released in response to these venoms can provide important insights into the pathophysiological mechanisms involved in snakebite accidents and contribute to the development of more effective treatments, such as antivenoms and inflammation modulators.

## 1. Introduction

The release of cytokines in the peritoneal fluid (PF) is a fundamental aspect of the inflammatory response, especially in experimental models that simulate acute or chronic inflammation. The peritoneum, a membrane that lines the abdominal cavity and the organs contained within it, is rich in immune cells such as mast cells, macrophages, neutrophils, and lymphocytes, which are rapidly activated in response to infectious, toxic, or traumatic stimuli [1,2].

After an inflammatory stimulus, these immune cells in the peritoneum release a series of cytokines, which are essential signaling molecules for communication between immune system cells [3,4]. Key cytokines involved in the early stages of inflammation include tumor necrosis factor-alpha (TNF-α), interleukin-6 (IL-6), IL-1β, IL-10, interferon-gamma (IFN-γ), chemokines like granulocyte-macrophage colony-stimulating factor (GM-CSF), monocyte chemoattractant protein-1 (MCP-1), and the mast cell degranulation marker mast cell protease-1 (MCPT-1). These molecules play a central role in leukocyte recruitment and the amplification of the inflammatory response, while anti-inflammatory cytokines such as IL-10 help regulate and resolve inflammation, promoting tissue recovery [5].

Studies involving the analysis of PF allow for the monitoring of cytokine release at different phases of inflammation, providing valuable information about the dynamics of local inflammatory responses. This type of investigation is relevant for understanding various inflammatory conditions such as infections and peritonitis and also for testing therapeutic interventions aimed at modulating inflammation and minimizing tissue damage [6].

The venoms of *Bothrops atrox* (Ba) and *Bothrops jararaca* (Bj) snakes are known to induce an intense inflammatory response in the body, being responsible for snakebite accidents that result in severe clinical manifestations. These venoms have a complex composition, rich in bioactive proteins such as snake venom metalloproteinases (SVMP), snake venom serine proteinases (SVSP), phospholipases A2 (PLA2), and lectins, which trigger a series of inflammatory and proteolytic events at the bite site [7,8,9,10]. Upon inoculation, these toxins promote tissue destruction, degradation of the extracellular matrix (ECM), and activation of immune system cells such as mast cells, neutrophils, and macrophages. This process results in the release of inflammatory mediators, such as pro-inflammatory cytokines and chemokines, which amplify the inflammation. Furthermore, the venoms increase vascular permeability, causing edema, hemorrhage, and intense pain [11]. *Bothrops jararaca* and *Bothrops atrox* are snakes of the Viperidae family of the Squamata order. *Bothrops jararaca* is commonly in the south-eastern region of Brazil, responsible for causing a wide range of snakebite accidents in the region and for its venom causing a broad inflammatory response, tissue damage, necrosis, hemorrhage, and death [12,13]. *Bothrops atrox* is found primarily in the Amazon, widely distributed in the region, and is the most clinically important snake in this area. This importance is not restricted to Brazil but extends to other countries in this region. However, despite its impact, the venom of this snake is not included in the set of immunogens used to produce antivenoms. In addition, some countries, such as Ecuador, do not have local production of antivenoms and are dependent on imports, which also do not include *Bothrops atrox* in their composition [9,14].

The release of cytokines in the peritoneal fluid of female mice after snakebites involving the snakes *Bothrops* jararaca and *Bothrops atrox* is still not entirely clear since male mice are often used in these experiments [15,16,17,18].

Female mice, like women, have distinct immune responses compared to males due to hormonal factors such as estrogen, which can modulate the release of cytokines, influencing the intensity of the inflammatory response. Studying female mice allows us to understand how biological sex affects the response to envenomation [19,20].

In this study, we investigated the cytokine profiles released in the peritoneal fluid of C57BL/6 female mice following injection with *Bothrops atrox* and *Bothrops jararaca* venoms. Our findings revealed that *Bothrops atrox* venom induced a significantly more intense inflammatory response compared to *Bothrops jararaca*, characterized by higher levels of TNF-α and MCP-1 in the peritoneal fluid. This heightened inflammatory response was associated with increased leukocyte recruitment and more pronounced tissue damage due to biochemical differences in the composition of the venoms.

This heightened inflammatory response was associated with increased leukocyte recruitment and more pronounced tissue damage. Understanding the cytokine dynamics in response to these venoms provides valuable insights into the pathophysiological mechanisms of snakebite envenomation and may contribute to the development of targeted therapeutic strategies, including improved antivenoms and inflammation modulators [7,16,21].

## 2. Results

### 2.1. Evaluation of the Electrophoretic and Enzymatic Profile of Venoms

**Figure 1:**
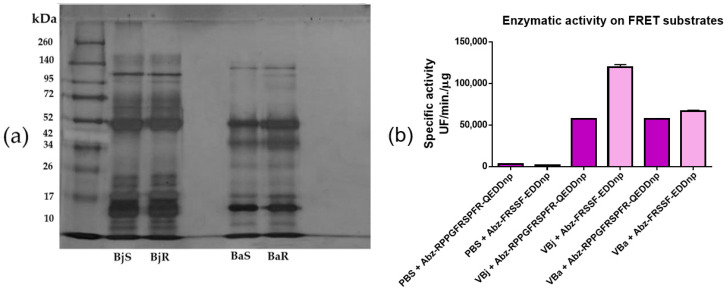
Electrophoretic profile of *Bothrops jararaca* and *Bothrops atrox* venom. A total of 5 µg of the venoms were subjected to polyacrylamide gel electrophoresis (12.5% for lower gel and 5% for upper gel) under reducing and non-reducing conditions. The bands were revealed by silver nitrate impregnation. BjS: *Bothrops jararaca* without reduction, BjR: *Bothrops jararaca* with reduction, BaxS: *Bothrops atrox* without reduction, BaxR: *Bothrops atrox* with reduction (**a**). Proteolytic activity of *Bothrops jararaca* and *Bothrops atrox* venom. In 96-well white bottom plates, 2.5 μL/well of each sample was incubated with 2.5 μL/well of the FRET substrates (Abz-Metalo and Abz-Serino) in PBS pH 7.4 (final volume of 100 μL/well). Proteolytic activity was measured by spectrofluorometric (λEX = 320 and λEM = 420 nm, reader: Hidex Sense, Hidex, Finland) for 15 min. Assay performed in duplicate (**b**). Representative data from two independent assays.

The analysis of the electrophoretic profile of the venoms of the snakes *Bothrops jararaca* and *Bothrops atrox* revealed the presence of proteins with molecular masses ranging from 8 to 145 kDa. The low-weight bands suggest the presence of dimers in the non-reduced sample (Figure 1a). The evaluation of enzymatic activity using 15-minute kinetics revealed the presence of snake venom metalloproteinases (SVMPs) and serine proteases (SVSPs) (Figure 1b). These results, together with the electrophoretic profile, confirm the presence of those toxins.

### 2.2. Evaluation of Pro- and Anti-Inflammatory Cytokines After 30% of the LD_50_ of Bothrops jararaca and Bothrops atrox Venoms

**Figure 2:**
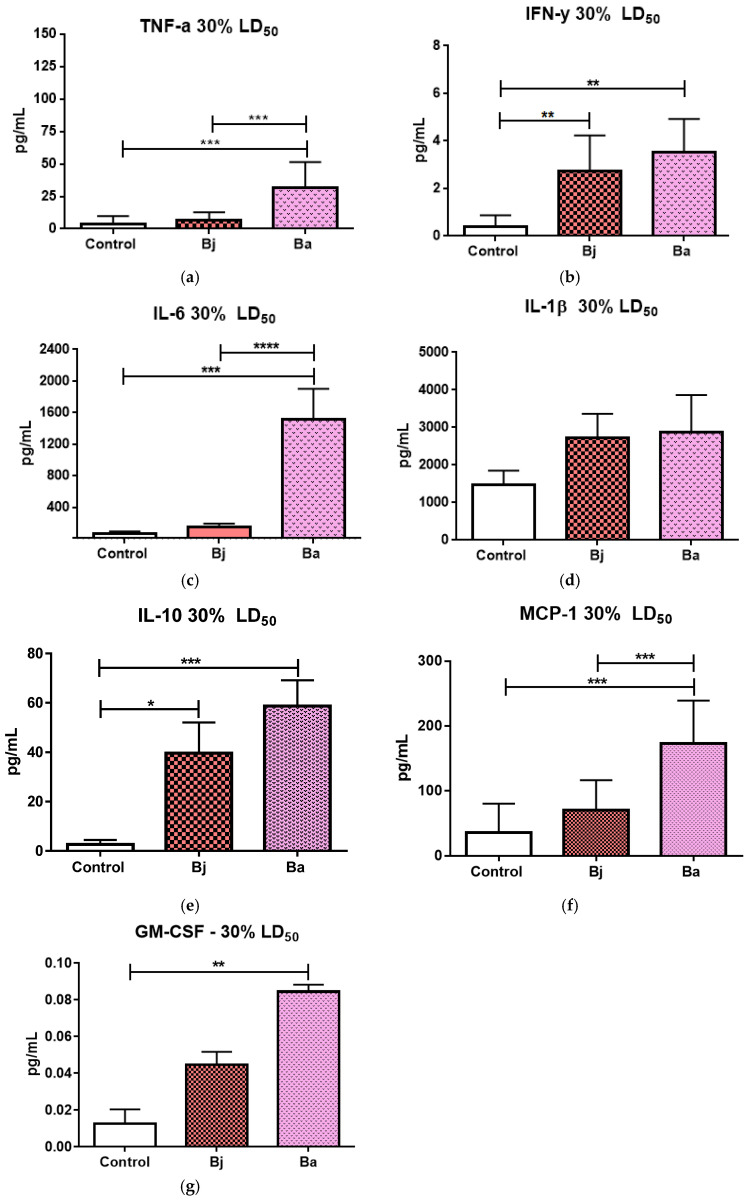
Quantification of chemokines and inflammatory cytokines present in the peritoneal fluid of mice after injection of 30% of the LD50 (n = 9/group) of the venoms of *Bothrops jararaca* and *Bothrops atrox*. TNF-α (**a**), IFN-γ (**b**), IL-6 (**c**), IL-1β (**d**), anti-inflammatory cytokine IL-10 (**e**); pro-inflammatories chemokines MCP-1 (**f**) and GM-CSF (**g**). Data analyzed statistically by One-way ANOVA followed by Dunnet’s post-test. (*) *p* < 0.05 statistically different; (**) *p* < 0.0019; (***) *p* < 0.0006; (****) *p* < 0.0001. Coefficient of variation: TNF-α: Control 192.70%, Bj 86.88%, Ba 62.52%; IFN-y: Control 122.35%, Bj 54.15%, Ba 40.38%; IL-6: Control 29.73%, Bj 25.24%, Ba 25.96%; IL-1β: Control 25.92%, Bj 23.22%, Ba 34.80%; MCP-1: Control 36.12%, Bj 70.54%, Ba 173.0%; GM-CSF: Control 63.04%, Bj 18.81%, Ba 24.53%. Control = control animal group with PBS, Bj: animals treated with *Bothrops jararaca* venom, Ba: animals treated with *Bothrops atrox* venom. Representative data from tests repeated three times.

Intraperitoneal injection of 30% of the lethal dose (LD50) of *Bothrops jararaca* and *Bothrops atrox* venoms significantly increased pro-inflammatory cytokines, such as TNF-α, IFN-γ, and IL-6, compared to the control group (PBS) (Figure 2a–c). No significant differences were observed in IL-1β levels (Figure 2d). The anti-inflammatory cytokine IL-10 was elevated in venom-treated groups compared to the control (Figure 2e). Additionally, a significant increase in chemokines MCP-1 and GM-CSF was observed in the group treated with *Bothrops atrox* venom, compared to both the control and the group treated with *Bothrops jararaca* venom (Figure 2f,g).

### 2.3. Evaluation of MCPT-1 Levels in Mice Treated with 30% of the LD_50_ of Bothrops jararaca and Bothrops atrox Venoms Using the ELISA Method

**Figure 3:**
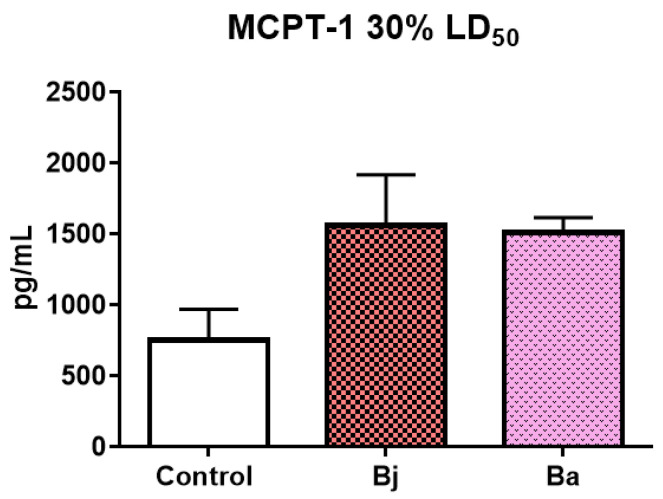
Quantification of the enzyme MCPT-1 present in the peritoneal fluid of mice (n = 9/group) post-injection of 30% of the LD50 of the *Bothrops jararaca* and *Bothrops atrox* venoms. One-way ANOVA followed by Dunnet’s post-test analyzed data statistically. Coefficient of variation: MCPT-1: Control 29.06%, Bj 22.86%, Ba 7.08%. Control = control group of animals with PBS, Bj: animals treated with the venom of *Bothrops jararaca* and Ba: animals treated with the venom of *Bothrops atrox*. Representative data from tests repeated three times.

Analysis of Mast Cell Protease-1 (MCPT-1) levels by ELISA showed an increase in the venom-treated groups compared to the control group, although the differences were not significant (Figure 3).

### 2.4. Mast Cell Activation Caused by 30% of the LD_50_ of Bothrops jararaca and Bothrops atrox Venoms

**Figure 4:**
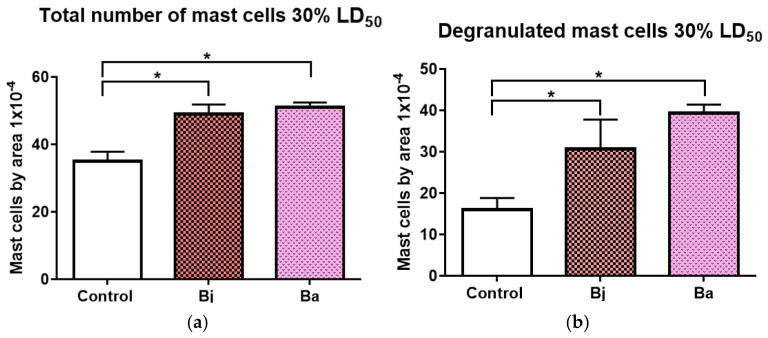
Effect of *Bothrops jararaca* and *Bothrops atrox* venoms on mast cell counts in peritoneal fluid. Total number of mast cells present in peritoneal fluid (**a**), Total number of degranulated mast cells in peritoneal fluid (**b**). One-way ANOVA followed by Dunnet’s post-test analyzed data statistically. (*) *p* < 0.05 statistically different. Coefficient of variation: Total number of mast cells: Control 8.08%, Bj 5.77%, Ba 2.77%; Degranulated mast cells: Control 17.68%, Bj 23.13%, Ba 5.29%. Control = control animal group with PBS, Bj: animals treated with *Bothrops jararaca* venom, and Ba: animals treated with *Bothrops atrox* venom. Representative data from tests repeated three times.

Cell counting was performed on the peritoneal fluid of mice following intraperitoneal injection of 30% of the LD50 of *Bothrops jararaca* and *Bothrops atrox*. A significant increase in the total number of mast cells was observed in the venom-treated animals compared to the PBS control group (Figure 4a). Additionally, the number of degranulated mast cells also showed a significant increase in the venom-treated animals compared to the control. These results suggest mast cell activation in response to the toxins from the administered venoms (Figure 4b).

### 2.5. Evaluation of Pro- and Anti-Inflammatory Cytokines After 50% of the LD_50_ of Bothrops jararaca and Bothrops atrox Venoms

**Figure 5:**
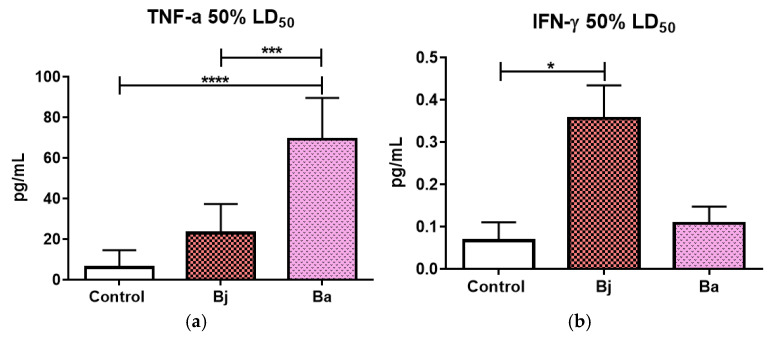

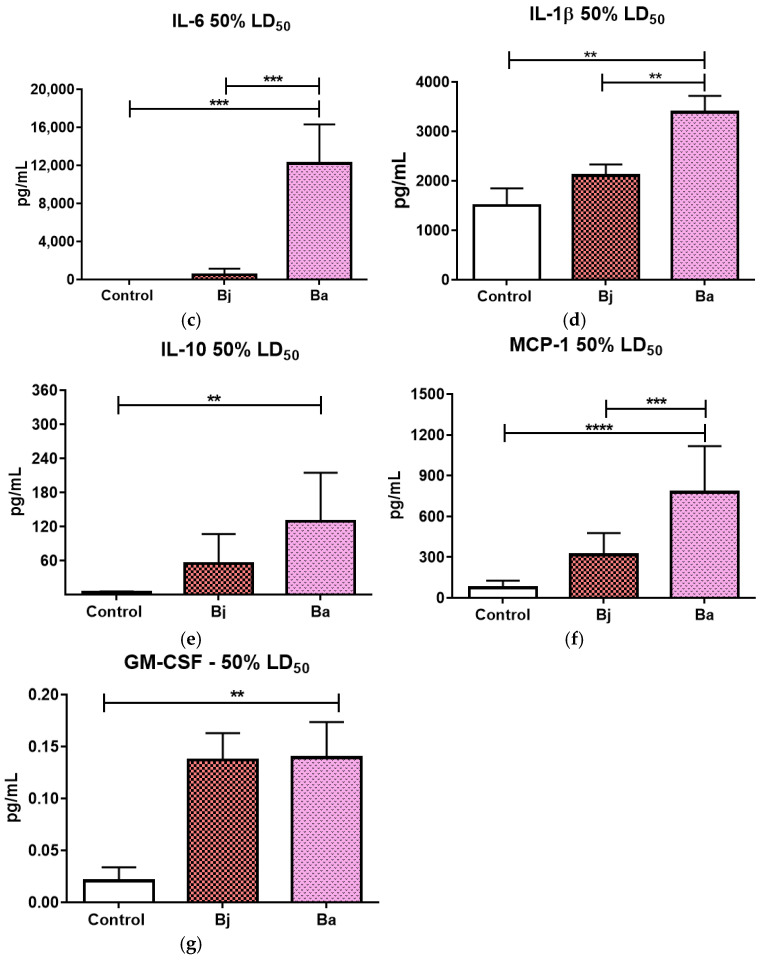
Quantification of chemokines and inflammatory cytokines present in the peritoneal fluid of mice after injection of 50% of the LD50 (n = 9/group) of the venoms of *Bothrops jararaca* and *Bothrops atrox*. TNF-α (**a**), IFN-γ (**b**), IL-6 (**c**), IL-1β (**d**), anti-inflammatory cytokine IL-10 (**e**); pro-inflammatories chemokines MCP-1 (**f**) and GM-CSF (**g**). Data analyzed statistically by One-way ANOVA followed by Dunnet’s post-test. (*) *p* < 0.05 statistically different; (**) *p* < 0.0019; (***) *p* < 0.0006; (****) *p* < 0.0001. Coefficient of variation: TNF-α: Control 140.88%, Bj 61.74%, Ba 29.49%; IFN-y: Control 185.16%, Bj 21.90%, Ba 126.57%; IL-6: Control 119.29%, Bj 127.06%, Ba 33.70%; IL-1β: Control 23.07%, Bj 10.50%, Ba 9.82%; IL-10: Control 40.19%, Bj 95.51%, Ba 66.62%; MCP-1: Control 67.60%, Bj 50.98%, Ba 43.65%; GM-CSF: Control 0.01245, Bj 0.0445, Ba 0.08435. Control = control animal group with PBS, Bj: animals treated with *Bothrops jararaca* venom, Ba: animals treated with *Bothrops atrox* venom. Representative data from tests repeated three times.

Tests using 50% of the LD50 of *Bothrops jararaca* and *Bothrops atrox* show us that *Bothrops atrox* venom significantly increased the release of pro-inflammatory cytokines TNF-α, IL-6, IL-1β (Figure 5a,c,d) and the anti-inflammatory cytokine IL-10 (Figure 5e). The *Bothrops* jararaca venom treatment showed a significant increase in IFN-γ expression compared to the control group (Figure 4b). Additionally, *Bothrops atrox* venom treatment led to a significant increase in MCP-1 and GM-CSF chemokines compared to both the control and the *Bothrops jararaca* venom groups (Figure 5f,g). Samples were diluted 1:2 using the assay diluent provided by the manufacturer.

### 2.6. Evaluation of MCPT-1 Levels in Mice Treated with Bothropic Venoms Using the ELISA Method

**Figure 6:**
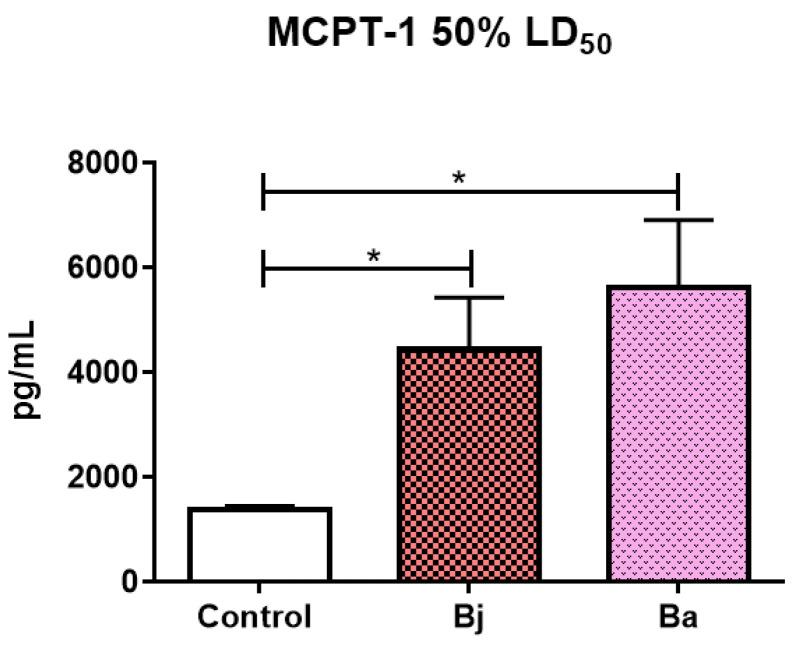
Quantification of the enzyme MCPT-1 present in the peritoneal fluid of mice (n = 9/group) post-injection of 50% of the LD50 of the *Bothrops jararaca* and *Bothrops atrox* venoms. One-way ANOVA followed by Dunnet’s post-test analyzed data statistically. (*) *p* < 0.05 statistically different. Coefficient of variation: MCPT-1: Control 5.20%, Bj 22.60%, Ba 22.90%. Control = control group of animals with PBS, Bj: animals treated with the venom of *Bothrops jararaca* and Ba: animals treated with the venom of *Bothrops atrox*. Representative data from tests repeated three times.

MCPT-1 levels were measured after injection of 50% of the LD50 of Bothrops jararaca and *Bothrops atrox* venoms. The enzyme levels showed an increase in the venom-treated groups compared to the control group. These results suggest mast cell activation in response to the venom toxins (Figure 6).

### 2.7. Mast Cell Activation Caused by 50% of the LD_50_ of Bothrops jararaca and Bothrops atrox Venoms

**Figure 7:**
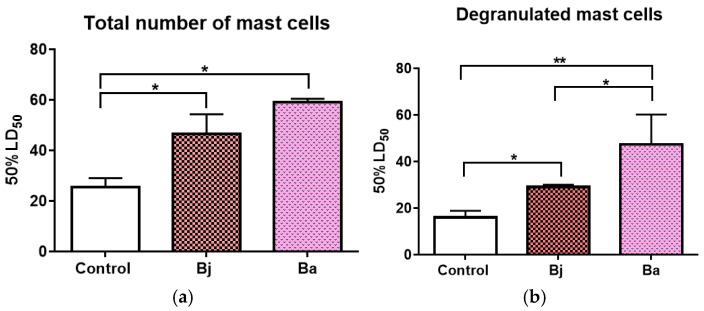
Impact of *Bothrops jararaca* and *Bothrops atrox* venoms on mast cells count in peritoneal fluid. Total number of mast cells present in peritoneal fluid (**a**), Total number of degranulated mast cells in peritoneal fluid (**b**). One-way ANOVA followed by Dunnet’s post-test analyzed data statistically. (*) *p* < 0.05 statistically different; (**) *p* < 0.0019. Coefficient of variation: Total number of mast cells: Control 13.86%, Bj 16.73%, Ba 2.40%; Degranulated mast cells: Control 17.68%, Bj 3.45%, Ba 27.17%. Control = control animal group with PBS, Bj: animals treated with *Bothrops jararaca* venom, and Ba: animals treated with *Bothrops atrox* venom. Representative data from tests repeated three times.

We counted cells in mice’s peritoneal fluid after they were given venom from *Bothrops jararaca* and *Bothrops atrox*. A significant increase in both total mast cell counts and degranulated mast cells was observed in the venom-treated animals compared to the PBS control group (Figure 7a). Furthermore, the group treated with *Bothrops atrox* venom showed a significantly higher number of degranulated mast cells compared to the *Bothrops jararaca* group (Figure 7b).

## 3. Discussion

Although we used female mice in this study to explore the potential hormonal influence on inflammatory responses, the data presented here do not provide a comprehensive analysis of this aspect. Although estrogen, a sex hormone, is known to modulate the immune response and may influence cytokine production [22,23,24,25,26,27], further studies and data are needed to better understand its specific role in the inflammatory response in our experimental model. This may allow for the analysis of more pronounced and specific immune responses, making the data on cytokine release more robust. In general, females tend to have more intense IN and AI responses than males, which may be relevant to understanding the dynamics of cytokine production during snakebite. Previous studies used males in experimental models of inflammation and envenomation, which facilitates comparison of the results obtained with the existing literature [16,19,20]. This methodological alignment may be crucial for validating or contrasting new data with previous studies, broadening the understanding of immune responses to snake envenomation. Thus, the choice of females for this study was a strategy to explore more deeply the interactions between snake venom and the immune system [15,16,17].

The results of this study provide important insights into the inflammatory effect of the venoms of *Bothrops atrox* (VBa) and *Bothrops jararaca* (VBj) that can emphasize the release of inflammatory cytokines in the peritoneal fluid of mice. The inflammatory response observed varied significantly between the two species of snakes and also due to the sublethal doses of venom (30% and 50% of the LD50). Tumor necrosis factor alpha (TNF-α) is a central cytokine in the acute inflammatory response, known to induce fever, apoptosis of infected or damaged cells, and recruitment of immune cells. The significant increase in TNF-α in response to VBa at both doses (30% and 50% of the LD50) suggests that this venom causes a more exacerbated inflammation compared to *Bothrops jararaca*.

VBa is known to contain a high concentration of metalloproteinases (SVMPs) and phospholipases A2 (PLA2s), which are enzymes with potent pro-inflammatory activity; these enzymes induce tissue damage, activation of immune cells, and release of inflammatory mediators [28,29]. Although VBj also contains SVMP and PLA2, the proportion and activity of these enzymes are different. The electrophoretic profile of VBj shows to be abundant in proteins with molecular weights between 10 and 17 kDa compared to VBa, suggesting that peptides such as bradykinins, which have vasodilatory and edematogenic effects, may not stimulate cytokine release as intensely as the enzymes present in VBa. These differences highlight the complexity and variability of the mechanisms of action of snake venoms, even among species of the same genus.

Furthermore, it has already been described in the literature that VBa results in a prominent inflammatory response with the influx of leukocytes characteristic of the acute inflammatory phase, justified by the release of vasoactive compounds induced by this venom [2,18,19,20,30]. The VBa, having a lower hemorrhagic potential when compared to VBj [31,32], can also explain this result. Additionally, the elevation of gamma interferon (IFN-γ) was another striking characteristic in the response to VBa, especially at the dose of 30% of the LD50. IFN-γ is a cytokine typically produced by T cells and natural killer (NK) cells, being crucial for macrophage activation and promotion of IN and Ad immune responses against intracellular pathogens. The observed increase in IFN-γ may indicate a robust activation of T cell-mediated immune pathways and suggests that VBA triggers a more intense and systemic inflammatory response [33,34]. Interestingly, at the 50% LD50 dose, the increase in IFN-γ was significant in the group treated with VBj but not in the group treated with Ba, which suggests a possible saturation of the immune response at the higher dose of VBa, while VBj-induced a more pronounced increase in IFN-γ under this condition. The increase in Interleukin-6 (IL-6), a key cytokine in regulating the inflammatory response and activation of IN systemic cells, was also observed in both groups treated with VBa. The VBj, although it induced an increase in IL-6, had a less pronounced response. IL-6 has a dual role: it is fundamental in the acute phase of inflammation, promoting the recruitment of neutrophils and the differentiation of T cells into Th17 cells, but it also exerts anti-inflammatory functions in later stages of inflammation. The intense IL-6 response induced by VBa indicates a potent activation of the inflammatory axis, which may be related to greater tissue damage and greater activation of innate immune pathways in response to this venom. Monocyte chemotactic protein 1 (MCP-1 or CCL2) plays an important role in the recruitment of monocytes, dendritic cells, and macrophages to sites of inflammation. The significant increase in MCP-1 in the groups treated with VBa, both at 30% and 50% of LD50, indicates strong recruitment of mononuclear cells to the site of inflammation. This effect was more pronounced compared to VBj, suggesting that VBa may cause greater recruitment of inflammatory cells to the injury site. This finding is consistent with the greater presence of metalloproteinases and phospholipases in VBa, which have already been described as inducers of chemokines such as MCP-1, promoting exacerbated tissue inflammation and facilitating cell infiltration [2,3,35].

IL-10 is a crucial anti-inflammatory cytokine to limit tissue damage caused by excessive immune responses. The increase in IL-10 observed mainly in the groups treated with Ba suggests that despite the strong inflammatory activation, there is also an active counter-regulation to minimize the impact of tissue damage [36,37,38,39]. This balance between pro-and anti-inflammatory cytokines is characteristic of immune responses induced by toxins, where the organism attempts to minimize excessive inflammation while combating the toxic effects of the venom. In contrast, the IL-10 response was less pronounced in the group treated with VBj, suggesting that this venom may generate a more controlled inflammatory response or that the activation of anti-inflammatory mechanisms may be less necessary due to the lower magnitude of the initial inflammatory response.

Granulocyte-macrophage colony-stimulating factor (GM-CSF) is known to promote the proliferation and activation of myeloid cells, such as neutrophils and macrophages. The increase in GM-CSF observed in the group treated with VBa indicates a robust activation of IN immune cells, which may be associated with the increased recruitment and activation of these cells at sites of tissue damage. The presence of GM-CSF may further amplify the inflammatory response, increasing the production of other inflammatory cytokines and contributing to the severity of the inflammatory picture induced by VBa [39,40].

The enzyme mast cell protease-1 (MCPT-1) is a marker of mast cell activation, cells that play a crucial role in the acute inflammatory response by releasing mediators such as histamine and cytokines. The increase in MCPT-1 in the groups treated with VBa and VBj indicates that both venoms are capable of activating mast cells, with the VBa seeming to induce a more pronounced activation. This activation of mast cells may contribute to the immediate symptoms of envenomation, such as pain, edema, and vasodilation, amplifying the local inflammatory response [6,9,18,40,41,42,43,44,45]. IL-1β is a pro-inflammatory cytokine that has a central role in inducing fever and promoting inflammatory processes. Interestingly, at the dose of 30% of the LD50, no significant differences were observed in the release of IL-1β, but with an increase in the dose to 50%, there was a significant increase in the groups treated with VBa. This result suggests that the release of IL-1β is dose-dependent, and that at higher sublethal doses, VBa may induce more severe tissue damage, leading to greater activation of IL-1β-mediated inflammatory pathways [39,40,41,42,43,44,45,46].

Further studies should be conducted to elucidate the precise mechanisms regulating the release of cytokines after the inoculation of VBa and VBj, as well as to investigate the variability in the immune response among different individuals and species. A better understanding of the possible interactions between the components of the venoms and the immune system could contribute to the development of more effective and specific therapies, minimizing the adverse systemic effects caused by snakebite accidents.

## 4. Conclusions

The results of this study have important implications for the clinical management of snakebite accidents. The difference in the immune response between the venoms of *Bothrops atrox* and *Bothrops jararaca* suggests that treatments should be adapted based on the species involved in the accident. The venom of *Bothrops atrox* induces a more pronounced inflammatory response, which may justify the more aggressive use of anti-inflammatory or immunomodulatory therapies, in addition to antivenom, to control the systemic effects of inflammation.

## 5. Materials and Methods

### 5.1. Venoms

The venoms of *Bothrops jararaca* (VBj) and *Bothrops atrox* (VBa) from the Brazilian territory were provided by the Herpetology Laboratory of the Instituto Butantan, obtained from adult male and female snakes, each measuring approximately 65 cm. For the assays, venom samples were dissolved in PBS pH 7.4 and stored in a freezer at −20 °C.

### 5.2. Analysis of the Electrophoretic Profile

Aliquots of each venom (*Bothrops jararaca* or *Bothrops atrox*) with the same concentration of 5 μg were treated according to the method described by Laemmli on 1970 and subjected to electrophoresis in SDS-polyacrylamide gel (SDS-PAGE) [46], using a concentration of 12.5% for the lower gel and 5% for the upper gel. After the run, the bands were revealed by impregnation with silver nitrate. The gel was photographed and then discarded.

### 5.3. Proteinase Activity Test

Proteolytic activity assays were performed in 96-well flat-bottomed white plates (Perkin Elmer, Waltham, MA, USA). The Fluorescent Resonance Energy Transfer (FRET) substrates Abz-RPPGFRSPFR-QEDDnp (Abz-Metalo, Uttar Pradesh, India), already defined as selective for SVMPs present in the venom of VBj and Dr. Fernanda Calheta Vieira Portaro, from the Laboratory of Structure and Function of Biomolecules, kindly provided Instituto Butantan VBax, and the substrate Abz-FRSSF-EDDnp (Abz-Serino, Jersey City, NJ, USA), already defined for SVSP. The tests were performed in a thermostabilized compartment (37 °C), and the reactions were continuously monitored (fluorescence emission at 420 nm after excitation at 320 nm) in a fluorimeter (Hidex Sense, Hidex, Finland). Proteolytic activity was expressed as mean reaction velocity (Vm). Experiments were performed in duplicate.

### 5.4. Animals

Female C57BL/6 mice (n = 27) aged 4 to 6 weeks weighing 18 to 20 g were provided by the Central Animal Facility of the Instituto Butantan. The animals were kept in the experimental animal facility of the Laboratory of Immunochemistry under standardized conditions, with water and food available ad libitum, according to certificate no. 2157260423 approved by CEUA IB, and following the guidelines established by CONCEA. The animals were divided into two groups for minimum and maximum doses (30% and 50%, respectively) of the LD50 values and subjected to intraperitoneal injection of each venom diluted in 200 μL of PBS. The animals in the control group were subjected to intraperitoneal injection of 200 μL of PBS pH 7.4. Each group contained 3 mice, and the tests were repeated three times. The LD50 values for the *Bothrops* group were kindly from the Technical Reports on In-Process Control of Venom and provided by Dra. Marisa Maria Teixeira da Rocha. After 30 min, the animals were euthanized by an overdose of the anesthetics Xylazine (30 mg/kg) and Ketamine (300 mg/kg); the abdomen was opened, and the peritoneal cavity was washed with 1 mL of cold, autoclaved PBS pH 7.4. After brief massage, the peritoneal exudate was collected using a 1 mL syringe and transferred to Eppendorf tubes. The exudate was centrifuged at 500× *g* for 10 min at 4 °C. The supernatant was stored in a −10 °C freezer for analysis of chemokines and inflammatory cytokines and inflammatory cytokines. Deposit-containing cells were suspended in PBS pH 7.4 and manually stirred; 100 µL collected, sprayed out on microscope slides, stained with toluidine blue, and counted in the Neubauer chamber.

### 5.5. Quantification of Chemokine and Inflammatory Cytokines

The chemokine and cytokine profile (MCP-1, TNF-α, IL-6, IFN-γ, and IL-10) induced by the venoms was evaluated in the cell-free peritoneal exudate using the “CBA Mouse Inflammatory Cytokines” kit (BD Bioscience, San Jose, CA, USA), according to the manufacturer’s specifications. Data acquisition was performed using a flow cytometer (BD FACSLyric^TM^, Milpitas, CA, USA), and analysis was conducted using CBA Analysis Software (BD) version 1.4. 

### 5.6. Quantification of IL-1β

The cytokine IL-1β was quantified from the supernatant samples obtained after exudate collection as described in item 5.4, using the ELISA method with the commercial kit “Mouse IL-1 beta Uncoated ELISA” (Invitrogen, Carlsbad, CA, USA), following the manufacturer’s recommendations. The reading was performed on a plate spectrophotometer (Cytation 3 Cell Imaging Reader, Biotek Instruments, Winooski, VT, USA) at a wavelength of 450nm. The lower limit of the kit’s standard curve was >1000 pg/mL.

### 5.7. Quantification of GM-CSF

The chemokine GM-CSF was quantified from the supernatants obtained after the collection of peritoneal exudates using the ELISA method with the commercial kit “Mouse GMCSF ELISA SET” (BD OptEIA^TM^, Franklin Lakes, NJ, USA), following the manufacturer’s recommendations. The reading was performed on a plate spectrophotometer (Cytation 3 Cell Imaging Reader, Biotek Instruments, Winooski, VT, USA) at a wavelength of 450 nm. The lower limit of the kit’s standard curve was >3000 pg/mL.

### 5.8. Quantification of MCPT-1

The enzyme MCPT-1, an important marker of mast cell degranulation and histamine release, was quantified from the supernatant samples obtained after exudate collection, as described in item 5.2, using the ELISA (Enzyme-Linked Immunosorbent Assay) method with the commercial kit “MCPT-1 (mMCP-1) Mouse Uncoated” (Chondrex and Invitrogen, Woodinville, WA, USA), following the manufacturer’s recommendations. The reading was performed on a plate spectrophotometer (Cytation 3 Cell Imaging Reader, Biotek Instruments, Winooski, VT, USA) at a wavelength of 540 nm. The lower limit of the kit’s standard curve was >15,000 pg/mL.

### 5.9. Statistical Analysis

The descriptive statistics of the results will be presented as mean ± standard error of the mean (SD). A one-way ANOVA statistical test was used. The test was followed by Dunnet’s post-test, with the *p*-value shown in the legend of the graph. Considering a significance criterion of *p* < 0.05, all statistical evaluations will be conducted using Prism GraphPad version 6.0.

## Data Availability

The original contributions presented in this study are included in the article. Further inquiries can be directed to the corresponding author(s).
